# All That Glitters Is Not Gold: Assessment of Bee Pollen Supplementation Effects on Gastric Mucosa

**DOI:** 10.3390/nu16010037

**Published:** 2023-12-21

**Authors:** Paweł Oszczędłowski, Kamil Górecki, Aleksandra Greluk, Milena Krawczyk, Katarzyna Pacyna, Jan Andrzej Kędzierawski, Artur Kacper Ziółko, Karol Chromiak, Mirosław A. Sławiński, Przemysław Raczkiewicz, Patrycja Chylińska-Wrzos, Barbara Jodłowska-Jędrych, Agnieszka Pedrycz-Wieczorska

**Affiliations:** 1 Students’ Scientific Association at the Department of Histology, Embryology and Cytophysiology, Medical University of Lublin, Radziwiłłowska 11, 20-080 Lublin, Poland61502@student.umlub.pl (K.P.);; 2 Department of Histology, Embryology and Cytophysiology, Medical University of Lublin, Radziwiłłowska 11, 20-080 Lublin, Poland; 3 Doctoral School, Medical University of Lublin, 20-093 Lublin, Poland

**Keywords:** bee pollen, flavonoids, dietary supplements, COX-1, COX-2, iNOS, ADMA, gastric mucosa

## Abstract

The aim of this study was to assess the influence of bee pollen supplementation on the levels of enzymes important for gastric mucosal homeostasis, namely cyclooxygenase-1 (COX-1), cyclooxygenase-2 (COX-2), inducible nitric oxide synthase (iNOS), and a biomarker—asymmetric dimethylarginine (ADMA)—in the gastric mucosa of Wistar rats. The experimental phase divided the rats into four groups: two control groups, sedentary and active, both not supplemented, and two experimental groups, sedentary and active, supplemented with bee pollen. The results indicated that bee pollen supplementation reduced the levels of COX-1 and elevated iNOS levels, while showing no significant impact on COX-2 levels. These findings do not conclusively support the gastroprotective and anti-inflammatory effects of bee pollen on gastric mucosa. However, the supplementation could have resulted in reduced ADMA levels in the physically active supplemented group. Our study does not unequivocally demonstrate the positive effects of bee pollen supplementation on the gastric mucosa, which may be attributed to the specific metabolism and bioavailability of substances within unprocessed, dried bee pollen. Further research should explore the topic of potential therapeutic applications of bee pollen in gastrointestinal health and its interactions with ADMA signaling pathways.

## 1. Introduction

Bee pollen (BP) is an apitherapeutic produced by forager bees in the process of creating bee bread [1]. It consists of their salivary secretions, nectar, and flower pollen grains, serving as an additional food source for the colony. The positive impacts of incorporating bee pollen into one’s diet have been proposed in ancient traditions and are supported by contemporary scientific research [2]. This has contributed to the enduring popularity of bee pollen as a dietary supplement in modern times [3]. Among its most numerous components are carbohydrates, proteins, lipids, fiber, ash, glucose, fructose, sucrose, and a wide array of macro- and microelements, as well as bioactive polyphenols, notably flavonoids [1]. Flavonoids are hydroxylated polyphenols containing at least one aromatic hydroxyl group and two or more aromatic rings linked by heterocyclic pyran. The wide range of biological features and favorable impact on human health have sparked a lot of interest in research on flavonoids in recent years [4]. It is suggested that some of the flavonoids, especially in the chalcones class, may possibly show gastroprotective potential. Among those, sofalcone has proven to fasten the rate of ulcer healing [5,6] by increasing stomach blood flow, enhancing gastric mucosa mucoprotein production, and affecting gastric tissue PG contents [7], as well as showing a direct bactericidal impact on *Helicobacter pylori* [8]. Quercetin, a flavonoid frequently found in bee products [9,10,11], has shown potential to protect gastric mucosa against ulceration [12]. It was also reported that flavonoids can increase the activity of antioxidant enzymes and the nuclear Nrf2 protein level in ulcerated gastric tissue, which protects cell membranes and promotes regeneration [13].

Cyclooxygenases (COX) consist of two isoforms, COX-1 and COX-2 [14]. They are closely related (they share >60% sequence identity) and catalyze the same reaction—the conversion of arachidonic acid to form prostaglandins, including PGD2, PGE2, PGF2α, prostacyclin (PGI2), and thromboxane [14,15,16,17]. COX-1 is constitutively expressed and it is involved in cytoprotective and regulatory functions in the gastrointestinal mucosa, platelets, kidneys, and the uterus [16]. However, it can be upregulated in endothelial cells by growth factors and shear stress [17]. In gastric mucosa, COX-1 is responsible for the synthesis of PGE2 and PGI2, which exert cytoprotective effects on several aspects of gastric function, such as an elevation in bicarbonate and mucus production, decrease in the secretion of gastric acid and pepsin, and the preservation of sufficient blood flow to the mucosa [17,18]. COX-2 stimulates inflammation in tissues [16,19]. COX-2 upregulation can even be linked to carcinogenesis [19,20,21]. Both COX-1 and COX-2 are expressed in gastric mucosa and inhibited by NSAIDs (non-steroidal anti-inflammatory drugs) [22,23]. NSAIDs are often used to inhibit the pro-inflammatory COX-2, which often results in the simultaneous inhibition of COX-1, which leads to the suppression of gastrointestinal-protective prostaglandin production. Conventional NSAID therapy can cause gastrointestinal mucosal injury, such as NSAID-related peptic ulcers and the associated serious complications of perforation, hemorrhage, and gastric obstruction [21,23,24]. COX-2 selective inhibitors (”coxibs”) reduce gastrointestinal side effects; however, prolonged usage is associated with cardiovascular side effects [20]. These conclusions further the need to investigate the properties of substances that may potentially inhibit COX-2, as well as their interference with COX-1 activity [19,20].

iNOS (inducible nitric oxide synthase) is an enzyme involved in the production of nitric oxide (NO). iNOS is an inducible isoform of the enzyme, which means its activity is triggered in response to inflammatory factors, toxins, infections, or other stimuli. It operates by converting the amino acid arginine into nitric oxide and citrulline using oxygen and NADPH. The generated NO has vasodilatory properties and plays a role in the body’s immune response to infections and inflammation [25]. Asymmetric dimethylarginine (ADMA) is an endogenous inhibitor of nitric oxide synthesis, competing with its substrate, L-arginine, which leads to inadequate nitric oxide levels in the blood, potentially causing harm to the body [26]. ADMA involvement in the pathogenesis of cardiovascular diseases and, ultimately, atherosclerosis has been well documented [27]. The ADMA/DDAH-1 signaling pathway has also recently been linked to gastric mucosal injury [28,29].

Both iNOS and COX-2 are elevated during inflammation, and their levels are controlled by the transcription factor NF-κB [30]. It was suggested that substances such as quercetin, rutin, and apigenin found in bee pollen can inhibit the levels of pro-inflammatory enzymes by their action on NF-κB, thereby showing their ability to limit the development of inflammatory response [30,31]. The extract of bee pollen’s polyphenols has also been found to reduce serum ADMA levels in mice [32]. Based on those findings, bee pollen supplementation could positively impact the health of gastric mucosa. Therefore, our study aimed to investigate the relationship between bee pollen supplementation on the levels of COX-1, COX-2, iNOS, and ADMA.

## 2. Materials and Methods

Our study protocol was the same as in the papers published by Jarosz et al. (2022) [33] and Zarobkiewicz et al. (2019) [34], as our research is a continuation of these studies, this time targeting bee pollen supplementation in the mucosa of the collected stomachs of the rats used in this study.

### 2.1. Animal Studies

This study included 30 eight-week-old rats. The rats averaged 330 g at the beginning of the study and about 400 g at the end. Twenty male Wistar rats were randomly divided into four equal groups: two sedentary (non-running)—control sedentary (Con-Sed, 1) and bee-pollen-supplemented sedentary (BP-Sed, 2)—and two running—control running (Con-Run, 3) and bee-pollen-supplemented (BP-Run, 4). In both levels of activity, one group was the control (Con-) and one was supplemented with bee pollen (BP-), as displayed in Table 1.

Multi-flower bee pollen from the vicinity of Lublin (Poland), collected in the period of July–August, was used in the study protocol. Bee pollen was not processed any further after its drying for preservation by the producer, as our study aimed to assess its effects in a state in which it is used by those who use it as a dietary supplement. In the whole experiment, there were two more groups of five rats supplemented with whey protein, as previously, bee pollen was reported to be of use against muscle degradation [35], although the results of whey-protein-supplemented groups are not in the scope of this paper. On average, 100 g of bee pollen contains approximately 23 g of protein, 31 g of carbohydrates, 5 g of lipids, a total of about 0.8 g of vitamins (A, E, D, B1, B2, B3, B5, B6, B7, C), and approximately 40 g water [36]. Approximately 13.4 g and 12 g of bee pollen were eaten per day by each rat of the BP-Run and BP-Sed group, respectively. Permanent access to water, standard feed, and bee pollen was provided. Food and supplements were weighted twice a day to determine how much was eaten each day per rat. During the 8-week laboratory phase, rats were weighed 15 times. All rats in running groups were running five times a week, 5 min each time, with a mean velocity of 6 km/h; no electrical shock was needed. Running was implemented, as consumers of bee pollen are usually followers of health- and environmentally conscious lifestyles [37]. Animals were cared for in accordance with the Guide for the Care and Use of Laboratory Animals [38]. The study protocol was approved by the First Local Ethical Committee for Experiments on Animals in Lublin (No. 24/2015), date of approval: 29 May 2015.

### 2.2. Laboratory Phase

After 8 weeks, all animals were decapitated, and their organs, including stomachs, were collected, formalin-fixed, and paraffin-embedded. Five micrometer-thick slides were prepared and used for standard histological staining with haematoxylin and eosin, as well as immunohistochemical (IHC) reactions for COX-1, COX-2, iNOS, and ADMA. Haematoxylin and eosin staining was performed using standard protocol [39]. Immunochemistry (IHC) was performed as previously described by Zarobkiewicz et al. (2019) and Jarosz et al. (2022) [33,34].

Monoclonal antibodies against COX-1 (catalog number: AF7002, Affinity Biosciences, Cincinnati, OH, USA), COX-2 (catalog number: AF7003, Affinity Biosciences, USA), iNOS (catalog number: AF0199, Affinity Biosciences, USA), and ADMA (catalog number: PAB301Ge01, CLOUD-CLONE CORP. (CCC, Santa Fe Springs, CA, USA),) were used. Antigenic sites were exposed to Proteinase K (Sigma-Aldrich, Saint Louis, MO, USA) for 5 min. Endogenous peroxidase activity was blocked by 0.3% solution of perhydrol in methanol. Non-specific binding was prevented by the addition of normal serum. The primary antibody was diluted as proposed by the manufacturer. The material was incubated with primary antibodies for 60 min, and afterwards, for another 30 min with HRP-conjugated secondary antibodies. The reaction was visualized with diaminobenzidine, and hematoxylin was used to counter-stain nuclei.

### 2.3. Data Analysis

Slides were evaluated under a light microscope. Sections were digitally photographed (Olympus BX-42 and CellSens Software V2.3). Digital images (magnification of ×400) were analyzed using image analysis software; ImageJ (Fiji, 2.9.0/14 September 2022) was used to manually count cells in IHC reactions and assess their reactivity (Scale: − (no visible reaction), + (visible normal levels of substance concentration), ++ (visible higher levels of substance concentration)). Evaluation of IHC results was made with the method previously applied by Lis-Sochocka et al. (2019); the sufficient number for the cell count was chosen for an antibody used in the IHC reaction based on the overall levels of concentration and reaction intensity in the slides, then cells were evaluated in different chosen areas of the slide [40]. Statistica 13 was used for statistical analysis. The Kruskal–Wallis test was used to verify the statistical significance of differences (in the distribution of cells into three levels of concentration) between groups. The level of significance was set as *p* < 0.05.

The study protocol cited above was as described previously, including detailed consumption data [33,34]. We have decided not to include the results of whey protein supplementation in this particular part of the study concerning the gastric mucosa, as their purpose in the whole experiment was to be compared with bee pollen groups in muscle analysis. What is more, whey protein protective effects on gastric mucosa are well known [41], and these results would not bring much novelty to this study.

## 3. Results

### 3.1. Histological Evaluation of Gastric Mucosa in Haematoxylin and Eosin Staining

**Figure 1:**
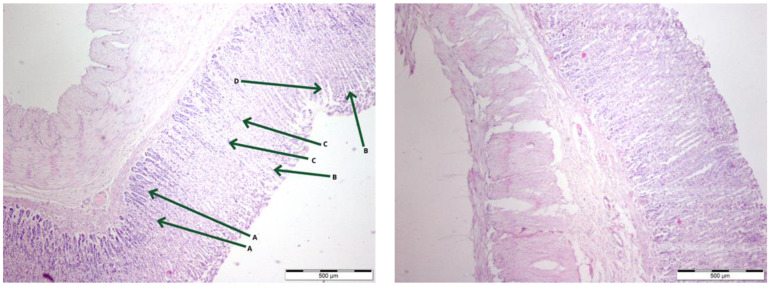
Comparison of gastric mucosa: Microscopic photographs of control group (Con-Run, 3) (**left**); bee-pollen-supplemented group (BP-Run; 6) (**right**). Displayed images show the architecture of the rat’s stomach with the emphasis on gastric mucosa. The image on the left shows where aforementioned structures are located: A—Parietal cells; B—Mucous cells; C—Chief cells; D—Gastric foveola, at the bottom of which is located the orifice of a gastric gland.

As expected, gastric mucosa was properly built in both control groups (Figure 1, left): visible chief cells were present, and distinct parietal cells with finely granular acidophilic cytoplasm and prominent nuclei were observed.

The gastric mucosa was appropriately structured in bee-pollen-supplemented groups (Figure 1, right). In the upper segments of the glands, mucous neck cells were present. Secretion was visible within the glandular lumen, in greater quantities compared to the control groups. However, a higher quantity of distinct chief cells was observed in the lower segments of the glands in comparison to the control groups. These cells were organized in clusters. Within the necks of the mucosal glands, numerous mucous neck cells were visible. Single parietal cells with finely granular acidophilic cytoplasm were observed. Illustrative images at 4x magnification depicting the physiological mucosal membrane and the mucosal membrane of the bee-pollen-supplemented group can be found in Figure 1.

### 3.2. Immunohistochemical Evaluation of COX-1, COX-2, iNOS, and ADMA Levels of Concentration

**Figure 2:**
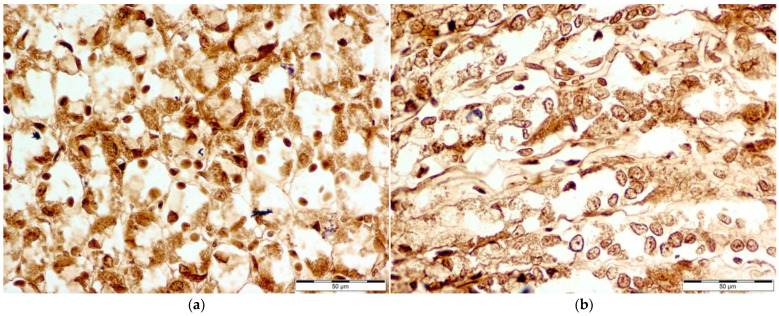
Comparison of COX-1 levels: Microscopic photography (**a**) control group (Con-Run, 3); (**b**) bee-pollen-supplemented group (BP-Run; 4). In both pictures, cytoplasmatic reaction seems to be comparable, although in the control group, there seem to be more deposits of antibodies linked to COX-1 around the nuclei of gastric cells, resulting in their darker tone and classification as cells with increased COX-1 levels.

**Figure 3:**
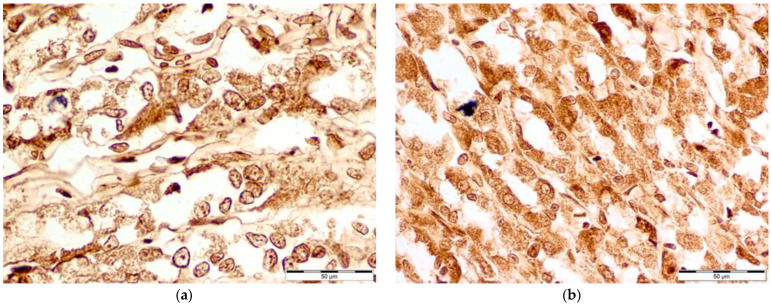
Comparison of COX-2 levels: Microscopic photography (**a**) control group (Con-Run, 3); (**b**) bee-pollen-supplemented group (BP-Run; 4). Concentration levels look similar in both photos, with almost equal distribution of pale, non-reactive cells, medium-toned cells that have shown standard levels of enzymes and the darkest cells with notable amounts of target-antibody complexes.

**Figure 4:**
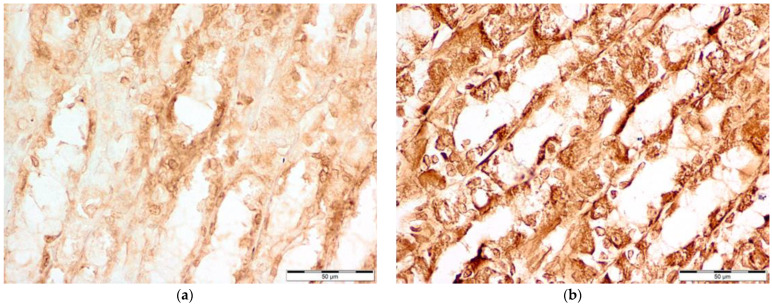
Comparison of iNOS levels: Microscopic photography (**a**) control group (Con-Run, 3); (**b**) bee-pollen-supplemented group (BP-Run; 4). While in the control group, the majority of cells show standard levels of the enzyme, with only a few cells that react strongly, and the majority of the cells in the image from the bee-pollen-supplemented group reacting with increased intensity. Such groups of strongly reacting cells were prevalent in slides from the bee-pollen-supplemented group, and have resulted in almost one-third of cells being labeled as (++).

**Figure 5:**
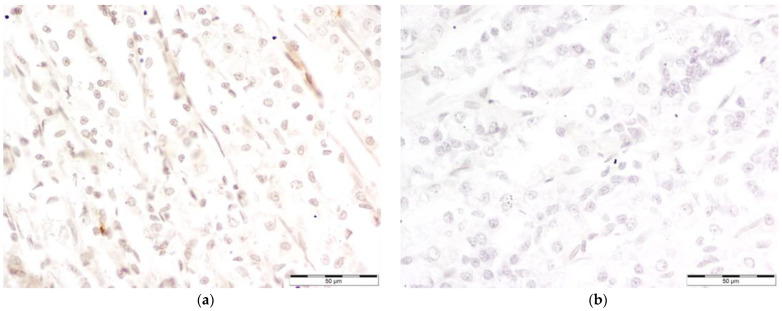
Comparison of ADMA levels: (**a**) control group (Con-Run, 3); (**b**) bee-pollen-supplemented group (BP-Run; 4). The almost uncolored image from bee-pollen-supplemented group indicates that in the majority of the cells, there was no immunohistochemical reaction. The color of nuclei shows only staining with hematoxylin, without any deposits from IHC. Cytoplasm of some cells is visible, with a slight coloring. These cells are labeled as positive in reaction, despite them being much weaker than reactions with enzymes in Figure 2, Figure 3 and Figure 4. Meanwhile, in the control group, it can be seen that some cells show not only positive reactions, but also a higher concentration represented by brown deposits of antibodies linked with ADMA molecules.

Microscopic photography id displayed in Figure 2, Figure 3, Figure 4 and Figure 5.

Cells observed in the microscopic images were classified into three groups according to the levels of a molecular marker confirmed through immunohistochemistry (IHC): (−) for the absence of the marker, (+) for detectable presence, and (++) for signs of heightened concentration. Information regarding the number of cells in each category in the reactions is provided in Table 2.

**Figure 6:**
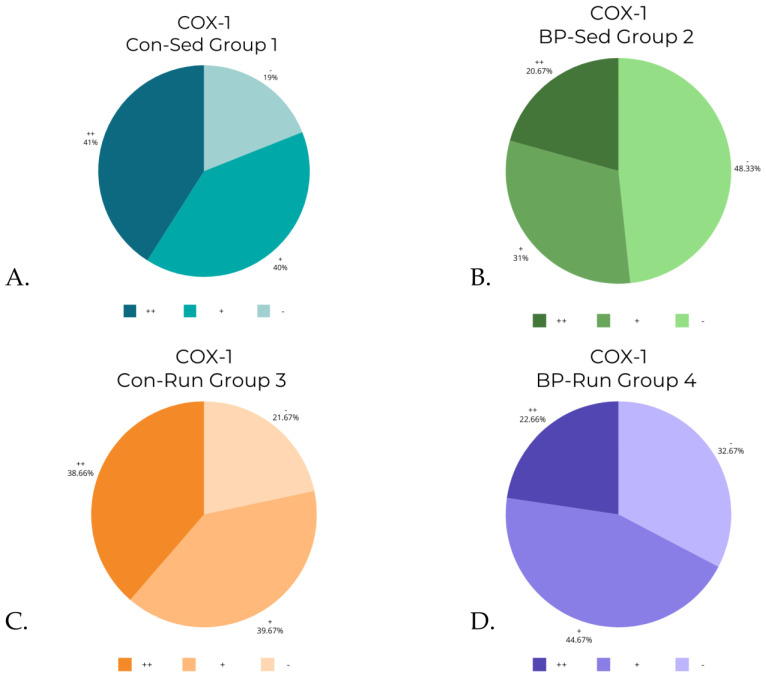
COX-1 levels in groups: (**A**) control, sedentary group of rats (Con-Sed; 1); (**B**) bee-pollen fed, sedentary group of rats (BP-Sed, 2); (**C**) control, running group of rats (Con-Run; 3); (**D**) bee-pollen fed, running group of rats (BP-Run, 4).

**Figure 7:**
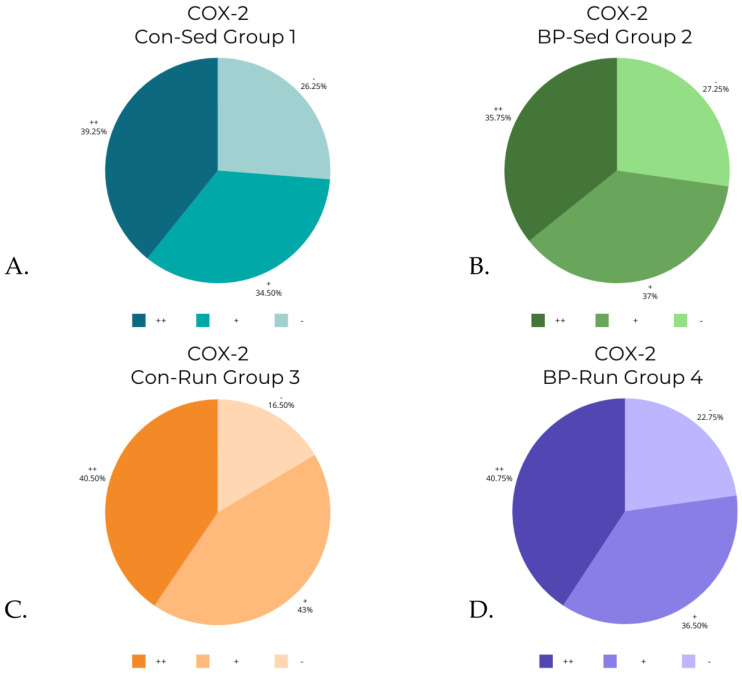
COX-2 levels in groups: (**A**) control, sedentary group of rats (Con-Sed; 1); (**B**) bee-pollen fed, sedentary group of rats (BP-Sed, 2); (**C**) control, running group of rats (Con-Run; 3); (**D**) bee-pollen fed, running group of rats (BP-Run, 4).

**Figure 8:**
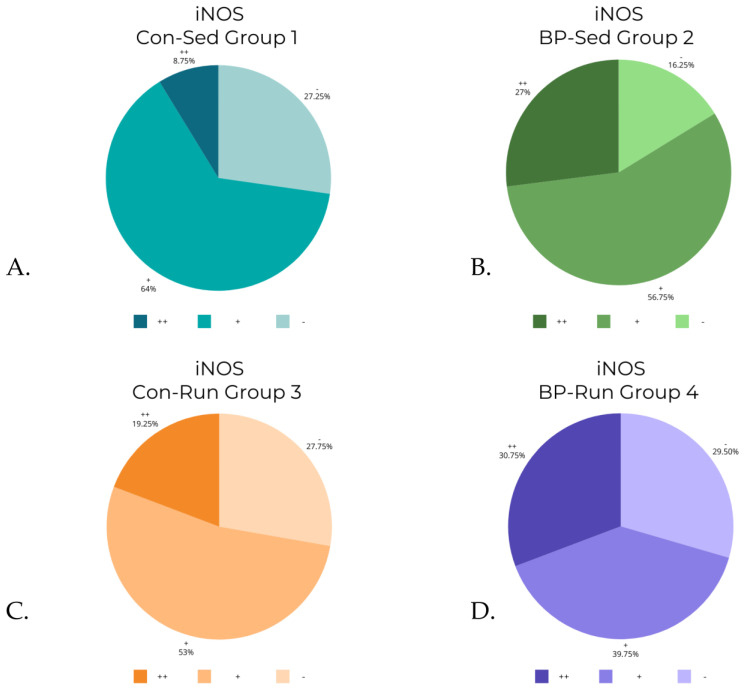
iNOS levels in groups: (**A**) control, sedentary group of rats (Con-Sed; 1); (**B**) bee-pollen fed, sedentary group of rats (BP-Sed, 2); (**C**) control, running group of rats (Con-Run; 3); (**D**) bee-pollen fed, running group of rats (BP-Run, 4).

**Figure 9:**
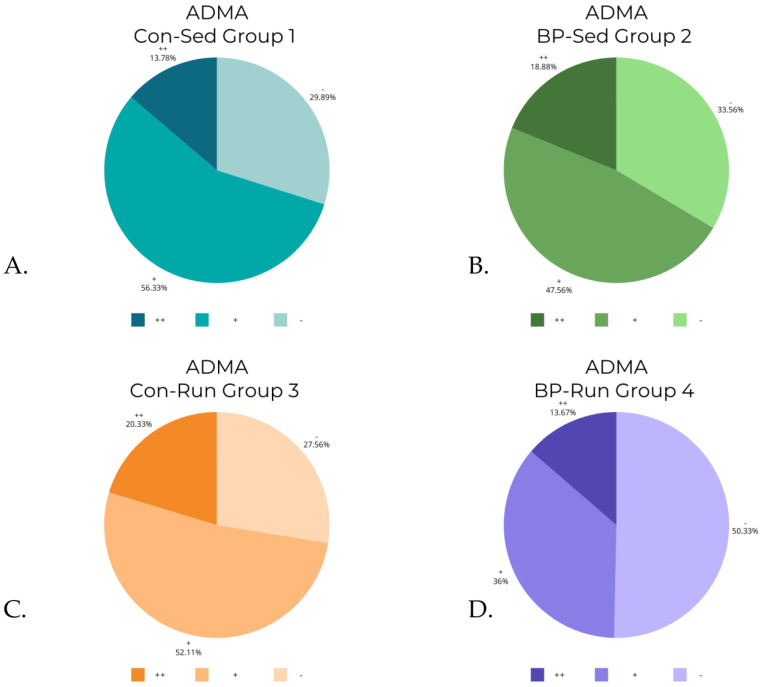
ADMA levels in groups: (**A**) control, sedentary group of rats (Con-Sed; 1); (**B**) bee-pollen fed, sedentary group of rats (BP-Sed, 2); (**C**) control, running group of rats (Con-Run; 3); (**D**) bee-pollen fed, running group of rats (BP-Run, 4).

The Kruskal–Wallis test revealed statistically significant differences in levels of COX-1 (*p* < 0.0001), iNOS (*p* < 0.0001), and ADMA (*p* < 0.001). A post hoc test revealed differences between groups (detailed reports are contained in Table 3, Table 4 and Table 5). The differences in COX-2 levels were almost at the border of the set *p* level, and were statistically significant in the Kruskal–Wallis test (*p* = 0.0486), although post hoc analysis revealed that there were no significant differences between groups. The data from Table 2 are visualized in Figure 6, Figure 7, Figure 8 and Figure 9.

### 3.3. COX-1 Levels

As shown in Table 2 and Table 3, levels of COX-1 were significantly lowered by bee pollen supplementation in both running and non-running groups compared to their control group (BP-Sed to Con-Sed and BP-Run to Con-Run). Physical activity had no impact on it in control groups, although it had lowered COX-1 levels in the BP-Run group compared to the BP-Sed group.

### 3.4. COX-2 Levels

Statistical analysis has revealed no statistically significant differences in levels of COX-2 between groups.

### 3.5. iNOS Levels

Corresponding Table 2 and Table 4 show that the levels of iNOS were heightened in bee-pollen-supplemented groups in both levels of activity, although the results were statistically significant only between sedentary groups (BP-Sed compared to Con-Sed).

### 3.6. ADMA Levels

The levels of this molecule were significantly lower than those of the previously described enzymes. Therefore, a significantly larger number of preparations subjected to IHC reaction was necessary to verify the results with sufficient precision (corresponding to a greater number of evaluated cells in Table 2). The levels of concentration significantly decreased only in the comparison between the running groups (BP-Run group compared to Con-Run). No statistically significant differences were observed between the other groups (Table 5).

## 4. Discussion

The potential of processed bee pollen in suppressing COX-2 and iNOS expression holds significant promise for the management of diverse health conditions, including gastric diseases. Inhibitors of cyclooxygenase-2 (COX-2) effectively mitigate the development of ulcers and upper gastrointestinal complications [42]. Furthermore, the COX-2–prostaglandin E2 pathway is directly associated with gastrointestinal carcinogenesis [43]. Levels of iNOS are increased in inflamed gastric tissue and rise along with oxidative stress [44]. Chronic inflammation promotes NO generation through iNOS, which is associated with the induction of neoplastic transformation [45]. The persistent and prolonged synthesis of nitric oxide (NO) by iNOS may be linked to direct interactions between NO and cellular components, leading to the generation of reactive nitrogen species that may potentially play a crucial role in the process of carcinogenesis [44]. Increased iNOS levels have been previously documented in individuals with peptic ulcers, proving the role of NO synthesized by iNOS in the pathogenesis of gastric ulcers [29]. Despite the fact that ADMA is primarily associated with cardiovascular diseases and is primarily utilized as a marker for them, it also holds significance in the pathophysiology of gastric ulcers [29,46]. Increased activity of the ADMA/DDAH-1 signaling pathway has been linked to the development of gastric mucosal damage [29].

In the study conducted by Lopes et al. (2019), it was revealed that bee pollen flavonoid extracts had an inhibitory effect on both COX isoforms [47]. What is interesting is that the used extract had a higher affinity for inhibiting the pro-inflammatory form of the enzyme, COX-2, and a lower affinity for the gastroprotective COX-1. Maruyama et al. (2010) reported that the ethanol extract of bee pollen produced from *Cistus* sp. of Spanish origin has shown its ability to inhibit COX enzymes and the production of NO. [48]. Bee pollen extract once again demonstrated its suspected anti-inflammatory activity by inhibiting the pro-inflammatory form of the enzyme COX-2 to a significantly greater extent than the constitutive COX-1. Additionally, it reduced the production of NO, a messenger molecule involved in the inflammatory response [48]. It was also previously described that some bee pollen flavonoid compounds (e.g., Quercetin and kaempferol) show the ability to not only inhibit COX enzymes but also to decrease the levels of pro-inflammatory COX-2 and iNOS [30,49]. Bee pollen extracts have also shown their ability to lower the plasmatic levels of ADMA in an animal model of a high-fat diet as a risk factor for cardiovascular disease [32,50].

In our study, the supplementation of bee pollen resulted in a reduction in COX-1 levels in both physically active and sedentary rats, while it had no statistically significant effects on COX-2 levels. Downregulation of COX-1 leads to a decrease in the synthesis of PGE2, which is known for its protective effect on the gastric mucosa [51]. What is more, in our study, bee pollen intake resulted in elevated levels of iNOS in the supplemented group. These results do not suggest the gastroprotective effects of bee pollen supplementation. Levels of ADMA seemed to be lowered by bee pollen supplementation compared to the control running group, which could be interpreted as a sign of mild gastroprotection, as ADMA is a marker of mucosal disorder in the stomach and a potential therapeutic target in mucosal injury treatment [52]. Although, it should be noted that this finding does not correspond with the rest of the results from our study and that physical exercise was found to diminish plasmatic levels of ADMA [46]. Further research should explore the topic of potential interaction between bee pollen flavonoids and the ADMA/DDAH-1 signaling pathway, preferably by measuring ADMA concentration quantitatively. The main limitation of our study is that histological methods, while being able to localize the changes in tissue, by design are unable to precisely measure exact concentrations. To the best of our knowledge, our study is one of the first to describe the influence of bee pollen supplementation on gastric mucosa, and the first to report the usage of unprocessed bee pollen on it.

What should be noted is that our study protocol had major differences when compared to the majority of the mentioned research on bee pollen’s properties. In our study, bee pollen was not processed in a laboratory, as we intended to use it as it is used by its consumers, in a ‘’pseudo-raw’’ state, in which it is not processed after being dried by the producer. In previously mentioned papers, bee pollen was administered in the form of purified extracts of bee pollen and bee pollen flavonoids (Maruyama et al., 2010, Lopes et al., 2019, Moita et al., 2013, Chelucci et al., 2023, Rzepecka-Stójko et al., 2017, Rzepecka-Stójko et al., 2018) [30,32,47,48,50,53].

Therefore, our research helps to fill a research gap about the effects of the consumption of bee pollen in a form that is easily accessible and usually consumed by dietary supplement users—dried bee pollen without any further processing. It was previously reported that bee pollen should be consumed fresh, as many of its nutritional properties are diminished in processes such as drying and lyophilization, where the temperature exceeds 40 °C [35,54]. What is more, pellets of bee pollen are covered in a tough pollen coat consisting of a cellulosic inner layer, intine, and an external layer, exine, made of sporopollenin [55]. This decreases pollen’s digestibility, often resulting in its passing through the intestinal tract without the absorption of its nutritional values [55]. Bee pollen can also contain various contaminants, such as pesticides, metals, mycotoxins, and pyrrolizidine alkaloids [37]. Finally, bee pollen’s chemical composition varies greatly, based on its place of origin, species of pollinator bee, and pollinated plant [35].

## 5. Conclusions

Taken together, the reasons stated above explain why the results of our research should not be considered as contradictory to the previous reports about the properties of bee pollen, but rather as complementary to them. Rather, they put more emphasis on why functional foods should be subject to control in terms of their actual active ingredient content and with regard to their processing methods and potential contaminants. Our results show no evident proof of gastroprotection nor anti-inflammatory and anti-oxidant effects of supplementation. Therefore, further research should be aimed at identifying methods of bee pollen processing that allow it to express its positive impact on stomach health; e.g., identification of specific flavonoid molecules that act as modulators of COX-1, COX-2, iNOS, and ADMA/DDAH-1 signaling pathways.

## Figures and Tables

**Table 1:** Groups of animals in the study design.

Group	Running	Supplementation	Group	Running	Supplementation
Con-Sed (1)	No	No	Con-Run (3)	Yes	No
BP-Sed (2)	No	Bee pollen	BP-Run (4)	Yes	Bee pollen

**Table 2:** Manually counted numbers of cells, grouped by level of concentration of the targeted molecule in IHC reaction (−)—absence of the marker, (+)—detectable presence, (++)—signs of heightened concentration (n)—number of cells.

Targeted Molecule	Group	− %	− (n)	+ %	+ (n)	++ %	++ (n)	Total (n) Evaluated
COX-1	1 (Con-Sed)	19.00%	57	40.00%	120	41.00%	123	300
	2 (BP-Sed)	48.33%	145	31.00%	93	20.67%	62	300
	3 (Con-Run)	21.67%	65	39.67%	119	38.66%	116	300
	4 (BP-Run)	32.67%	98	44.67%	134	22.66%	68	300
COX-2	1 (Con-Sed)	26.25%	105	34.50%	138	39.25%	157	400
	2 (BP-Sed)	27.25%	109	37.00%	148	35.75%	143	400
	3 (Con-Run)	16.50%	66	43.00%	172	40.50%	162	400
	4 (BP-Run)	22.75%	91	36.50%	146	40.75%	163	400
iNOS	1 (Con-Sed)	27.25%	109	64.00%	256	8.75%	35	400
	2 (BP-Sed)	16.25%	65	56.75%	227	27.00%	108	400
	3 (Con-Run)	27.75%	111	53.00%	212	19.25%	77	400
	4 (BP-Run)	29.50%	118	39.75%	159	30.75%	123	400
ADMA	1 (Con-Sed)	29.89%	269	56.33%	507	13.78%	124	900
	2 (BP-Sed)	33.56%	302	47.56%	428	18.88%	170	900
	3 (Con-Run)	27.56%	248	52.11%	469	20.33%	183	900
	4 (BP-Run)	50.33%	453	36.00%	324	13.67%	123	900

**Table 3:** COX-1: the *p*-value for multiple comparisons (two-sided) in post hoc tests after statistical significance was revealed in Kruskal–Wallis test (*p* < 0.0001).

Groups Compared	Con-Sed (1)	Con-Run (3)	BP-Sed (2)	BP-Run (4)
Con-Sed (1)		1.000000	<0.000001 ^1^	0.000009
Con-Run (3)	1.000000		<0.000001	0.000272
BP-Sed (2)	<0.000001	<0.000001		0.073937
BP-Run (4)	0.000009	0.000272	0.073937	

^1^ The level of statistical significance was set at *p* < 0.05, and such values are highlighted in red in the table.

**Table 4:** iNOS: the *p*-value for multiple comparisons (two-sided) in post hoc tests after statistical significance was revealed in Kruskal–Wallis test (*p* < 0.0001).

Groups Compared	Con-Sed (1)	Con-Run (3)	BP-Sed (2)	BP-Run (4)
Con-Sed (1)		0.405873	<0.000001 ^1^	0.001928
Con-Run (3)	0.405873		0.001687	0.460279
BP-Sed (2)	<0.000001	0.001687		0.375597
BP-Run (4)	0.001928	0.460279	0.375597	

^1^ The level of statistical significance was set at *p* < 0,05, and such values are highlighted in red in the table.

**Table 5:** ADMA: the *p*-value for multiple comparisons (two-sided) in post hoc tests after statistical significance was revealed in Kruskal–Wallis test (*p* < 0.001).

Groups Compared	Con-Sed (1)	Con-Run (3)	BP-Sed (2)	BP-Run (4)
Con-Sed (1)		0.129412	1.000000	<0.000001 ^1^
Con-Run (3)	0.129412		0.164388	<0.000001
BP-Sed (2)	1.000000	0.164388		<0.000001
BP-Run (4)	<0.000001	<0.000001	<0.000001	

^1^ The level of statistical significance was set at *p* < 0,05, and such values are highlighted in red in the table.

## Data Availability

Data are contained within the article.
